# Purification of *Houttuynia cordata* Thunb. Essential Oil Using Macroporous Resin Followed by Microemulsion Encapsulation to Improve Its Safety and Antiviral Activity

**DOI:** 10.3390/molecules22020293

**Published:** 2017-02-15

**Authors:** Jianmei Pang, Wujun Dong, Yuhuan Li, Xuejun Xia, Zhihua Liu, Huazhen Hao, Lingmin Jiang, Yuling Liu

**Affiliations:** 1 State Key Laboratory of Bioactive Substance and Function of Natural Medicines, Institute of Materia Medica, Chinese Academy of Medical Sciences & Peking Union Medical College, 1 Xiannongtan Street, Beijing 100050, China; pangjianmei@imm.ac.cn (J.P.); dwujun@imm.ac.cn (W.D.); xjxia@imm.ac.cn (X.X.); liuzhihua0207@163.com (Z.L.); haohuazhen@imm.ac.cn (H.H.); 2 Institute of Medicinal Biotechnology, Chinese Academy of Medical Sciences & Peking Union Medical College, Beijing 100050, China; yuhuanlibj@126.com; 3 Beijing Wehand-Bio Pharmaceutical Company Limited, Beijing 102600, China; jianglm4312@163.com

**Keywords:** *Houttuynia cordata* Thunb., essential oil, purification, microemulsion, safety evaluation, antiviral activity

## Abstract

Essential oil extracted from *Houttuynia cordata* Thunb. (*H. cordata*) is widely used in traditional Chinese medicine due to its excellent biological activities. However, impurities and deficient preparations of the essential oil limit its safety and effectiveness. Herein, we proposed a strategy to prepare *H. cordata* essential oil (HEO) safely and effectively by combining the solvent extraction and the macroporous resin purification flexibly, and then encapsulating it using microemulsion. The extraction and purification process were optimized by orthogonal experimental design and adsorption-desorption tests, respectively. The average houttuynin content in pure HEO was then validated at 44.3% ± 2.01%, which presented a great potential for industrial application. Subsequently, pure HEO-loaded microemulsion was prepared by high-pressure homogenization and was then fully characterized. Results showed that the pure HEO-loaded microemulsion was successfully prepared with an average particle size of 179.1 nm and a high encapsulation rate of 94.7%. Furthermore, safety evaluation tests and in vitro antiviral testing indicated that the safety and activity of HEO were significantly improved after purification using D101 resin and were further improved by microemulsion encapsulation. These results demonstrated that the purification of HEO by macroporous resin followed by microemulsion encapsulation would be a promising approach for industrial application of HEO for the antiviral therapies.

## 1. Introduction

Nowadays, there is a renewed interest in medicinal plants because of the potentially important sources of bioactive substance that may be very important in the field of medicine. Essential oils (EOs) are volatile, natural, complex compounds isolated from natural plant materials (flowers, buds, seeds, leaves, root, and stem). EOs have a broad spectrum of biological activities, such as anti-microbial [1,2,3], antiviral [4] and anti-inflammatory effects [5]. *Houttuynia cordata* Thunb. (*H. cordata*) injection, in which EO is the main active compound, is widely used to treat respiratory infections, conjunctivitis, keratitis, acute, chronic rhinitis, and sinusitis in traditional Chinese medicine therapy. Moreover, *H. cordata* injection was recognized as the formula for the prevention of Severe Acute Respiratory Syndrome (SARS) by the Health Ministry of China during the outbreak of the SARS epidemic [6]. However, the *H. cordata* injection was suspended for its severe adverse drug reactions (ADR), such as breathing problems, high fever, rash, anaphylactic shock, and even sudden death by the China Food and Drug Administration (CFDA) in 2006.

**Figure 1:**
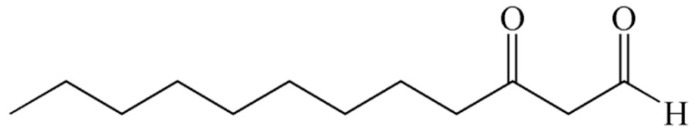
Chemical structure of Hou (3-oxododecanal).

Generally, *H. cordata* essential oil (HEO) is extracted from fresh *H. cordata* using hydrodistillation extraction and then dissolved in Tween 80 solution to prepare the *H. cordata* injection [7,8,9,10]. The volatile compounds include houttuynin (3-oxododecanal, Hou, Figure 1), 2-undecanone, pinene, camphene, myrcene, limonene, and so on, among which Hou is the main active compound [11]. However, it was reported that the Hou content in the injection was especially low (<1%, and even undetectable) [12]. Studies that investigated the relationship between ADR and Hou content showed that Hou is easily destroyed (oxidation, polymerization, or condensation reaction) by the high temperature during the hydrodistillation extraction due to its poor stability [13,14]. In addition, our group found that degradation products of Hou showed serious acute toxicity, which would induce ADR. *H. cordata* injection can also cause hemolysis, which may result from impurities of HEO or Tween 80 in the formulation [15,16]. Additonally, impurities (vegetable proteins, peptide polysaccharides, and tannins) in HEO may stimulate the immune system to produce ADR-like allergic reactions [15]. Therefore, a facile and comprehensive approach to avoid the occurrence of ADR is urgently needed.

Xue et al. reported that solvent extraction performed at room temperature is highly efficient to extract Hou-riched HEO [17]. Compared to the hydrodistillation extraction, the mild solvent extraction condition can avoid the destruction of Hou. However, they did not study the safety and activity of the extract. In addition, impurities in the extract were not removed, which were not conducive to the safety of the product. Therefore, purification is especially needed to remove impurities and further improve the safety and activity. However, to our knowledge, there is no study reported on purification of essential oil from *H. cordata*. Generally, liquid–liquid extraction [18], fractionation [19], and silica gel column chromatography [20] are used for purification of natural products. Unfortunately, these extraction procedures are inefficient, costly, time and solvent consuming, and labor intensive [21]. In addition, it is very difficult to enrich the essential oil due to its extremely low polarity and stability. Comparatively, macroporous resins (MRs) have unique advantageous adsorption properties due to the ideal pore structure and various surface functional groups available on these resins [22,23]. They are durable nonpolar, middle-polar, and polar macroporous polymers and have been successfully applied for the separation of flavonoids [24,25], saponins [26,27] and alkaloids [28] from traditional Chinese medicine or herbs. Moreover, MRs are relatively low-cost and easily regenerated, which is especially important for industrial applications.

Intravenous microemulsions (MEs) are biodegradable, biocompatible, physically stable, easy to scale up, and cost effective [29]. Most importantly, the good solubility in oil and lipophilicity of HEO make it possible to be encapsulated in the inner oil phase of MEs, thereby improving its stability and safety. This study aimed to develop a safe and highly active HEO preparation through purification by MR followed by ME encapsulation. In addition, safety evaluation tests and in vitro antiviral tests were performed with different extracts and preparations. We hope that this study will be helpful for future antiviral therapies and infectious diseases.

## 2. Results and Discussion

### 2.1. Solvent Extraction of Crude HEO

The extraction ratio of HEO was affected by many factors, such as solvent polarity, extraction time, root length, and solid-liquid ratio. Among them, the solvent is the main factor that determines the composition of extracts. In order to obtain HEO with high Hou content and narrow down the range of extraction parameters, optimization of solvents with different polarity were optimized before orthogonal tests. Here, solvents including non-polar petroleum ether (boiling range of 60–90 °C), medium polar ethyl acetate, and polar 95% ethanol were chosen for extraction using the Hou content as the index. Results showed that ethyl acetate had the highest Hou extract efficiency. Therefore, ethyl acetate was used as the solvent for the extraction.

To further optimize the extraction conditions, L9 (3) ^4^ orthogonal tests were employed and the experimental design was shown in Table 1 and Table 2. Table 1 showed the levels of each factor which would affect the extraction ratio of HEO. In Table 2, *k_i_* was the mean extraction ratio of the corresponding level of each factor and *R* was the range of the *k_i_* of each factor [30,31]. The parameter of *k_i_* was used for judging the optimal level of each factor while R was used for judging the effect of the factors. The results indicated that the optimized extraction condition was A2 (factor A level 2)/B3/C3/D2 and the effect of the factors was D > A > B > C. Therefore, sufficient extraction solvents with proper polarity could easily infiltrate to the herbs thereby improve the extraction efficiency. Additionally, ultrasonic time could improve the extraction efficiency and feasible extraction time and root length were beneficial for the penetration of solvent. Overall, the optimized extraction condition was as follows: the ultrasonic time was 20 min (A2), the extraction time was three days (B3), the *H. cordata* root length was 1.0 cm (C3), and the solid-liquid ratio was 1/1.5 (kg/L, D2). Using the optimized extraction condition, the HEO extraction ratio was 0.171% (wt %) and the Hou content in the extract was 8.80% (wt %) based on the GC analysis. Moreover, the extract was treated by alcohol sinking and *n*-hexane extraction to prepare the crude HEO (Hou 15.0%) for MR purification.

### 2.2. Optimization of Operational Parameters 

#### 2.2.1. Optimization of MR Types

**Figure 2:**
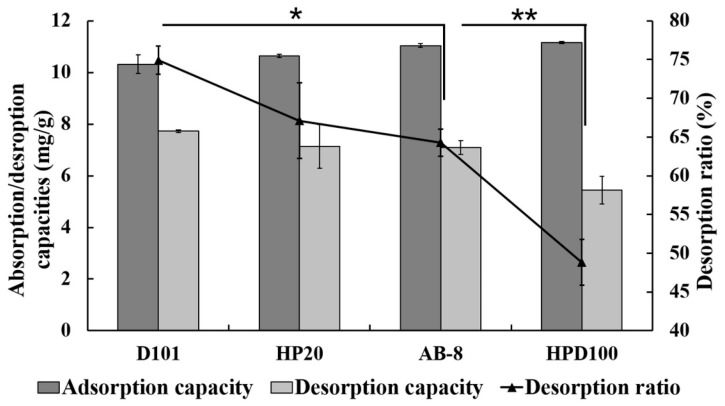
Adsorption and desorption capacities as well as desorption ratios of Hou on different MRs. Desorption ratios was evaluated statistically, * *p* < 0.05, ** *p* < 0.01 compared to D101.

MRs were selected in accordance with the structures and polarities of the adsorbed substances and resins [32]. As shown in Figure 1, Hou contains a non-polarity long aliphatic chain and a mid-polarity carbonyl structure, which indicating a weak polarity. Here, four types of MRs ranging from non-polar to mid-polar were screened for the adsorption and desorption tests. Their absorption capacities, desorption capacities and desorption ratios are shown in Figure 2. Results showed that all of the tested MRs had similar and high adsorption capacities (>10 mg/g). HPD100 had the highest adsorption capacity of 11.16 mg/g owing to its relative high surface area. As for desorption ratios, D101 resin showed the highest desorption ratio (74.9%) and was significantly higher than AB-8 and HPD100. This could be explained by the appropriate polarity of D101 and its larger pore diameters [33]. Considering the adsorption capacities and its corresponding desorption ratios, D101 was selected as the optimal resin for further purification.

#### 2.2.2. Optimization of the Adsorption Time 

**Figure 3:**
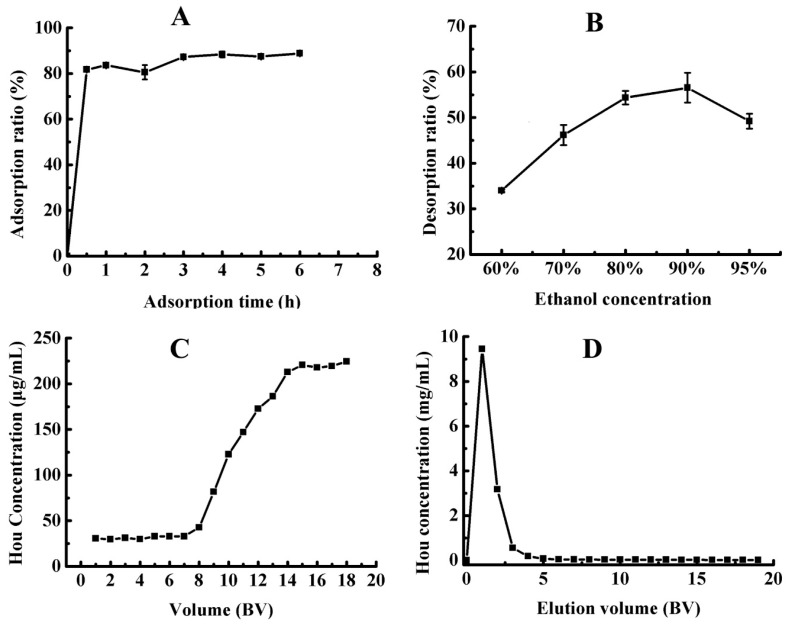
Adsorption kinetic curve of Hou on D101 (**A**); effect of different ethanol concentrations on Hou desorption ratio (**B**); dynamic breakthrough curve (**C**); and dynamic desorption curve (**D**) of Hou on D101. Note: BV: bed volume.

Adsorption time is an important factor affecting the adsorption ratio of Hou on D101. The adsorption kinetic curve is one of the important characteristics that define the adsorption efficiency. As shown in Figure 3A, the adsorption ratio of Hou on D101 increases rapidly in the first 30 min and then increases slowly and finally reaches the equilibrium after 3 h. The fast adsorption process of D101 resin in the first 30 min was due to the high diffusivity of Hou into macropores of the resin and the slow adsorption process 30 min later was because of its high transfer resistance into macropores. The result indicated that Hou exhibited a fast adsorption feature on D101 resin and 3 h was appropriate as the adsorption time.

#### 2.2.3. Optimization for the Concentration of the Ethanol Solution

Ethanol with low cost and nontoxic is usually used as the desorbent. Here, different concentrations of ethanol–water solutions (60:40, 70:30, 80:20, 90:10, and 95:5 (*v*/*v*)) were used to establish the proper desorption conditions. As shown in Figure 3B, the desorption ratio is the highest when the ethanol concentration was 90%. Thus, ethanol–water (90:10, *v*/*v*) solution was selected as the appropriate desorption solution and used in the following dynamic desorption experiments.

#### 2.2.4. Optimization for Breakthrough Volume

The breakthrough volume is important in solid phase extraction because it represents the sample mount that can be adsorbed on resin without loss of analytes during the loading process [34]. Generally, 10% ratio of the exit to the inlet solute concentration is defined as the breakthrough point. When the adsorption reaches the breakthrough point, the adsorption effect decreases, even disappears, and the analytes leak from the resin [35]. The dynamic breakthrough curve on D101 resin was obtained according to the volume of loading sample and the Hou concentration of the leak solution. As shown in Figure 3C, the Hou concentration in the exit solute was very low before 7 bed volume (BV) at the sample flow rate of 6 BV/h, which suggested that Hou was almost completely absorbed by D101 resin. Subsequently, the Hou concentration increased rapidly in the leak solution until it reacheed a steady plateau at 14 BV. The result indicated that the breakthrough volume of Hou on D101 resin was approximately 7 BV. Therefore, 7 BV of sample solution (Hou concentration: 0.225 mg/mL) was determined as the saturated adsorption volume for dynamic adsorption on D101 resin.

#### 2.2.5. Optimization for the Eluent Volume

Before Hou desorption, 2 BV of distilled water and 5 BV of ethanol-water (30:70, *v*/*v*) were used to flush the resin to remove the high polar components and impurities. Subsequently, ethanol–water (90:10, *v*/*v*) was used as the desorption solution and the dynamic desorption curve was obtained according to the eluent volume of ethanol–water (90:10, *v*/*v*) and the Hou concentration of the eluent. As shown in Figure 3D, the dynamic desorption curve was sharp and narrow with no trailing peak, which indicated that Hou was completely desorbed from D101 resin and enriched in approximately 3 BV of the eluent. After removing the solvent, pure HEO was obtained.

According to the above results, the optimal purification conditions were confirmed as follows: D101 resin was used to adsorb 7 BV of sample solution (Hou 0.225 mg/mL) for 3 h at a flow rate of 6 BV/h. Then, 2 BV of deionized water and 5 BV of 30% ethanol were used to remove the high polar components and impurities. At last, 5 BV of 90% ethanol was used for Hou desorption at a flow rate of 6 BV/h.

### 2.3. Analysis of Extract

**Figure 4:**
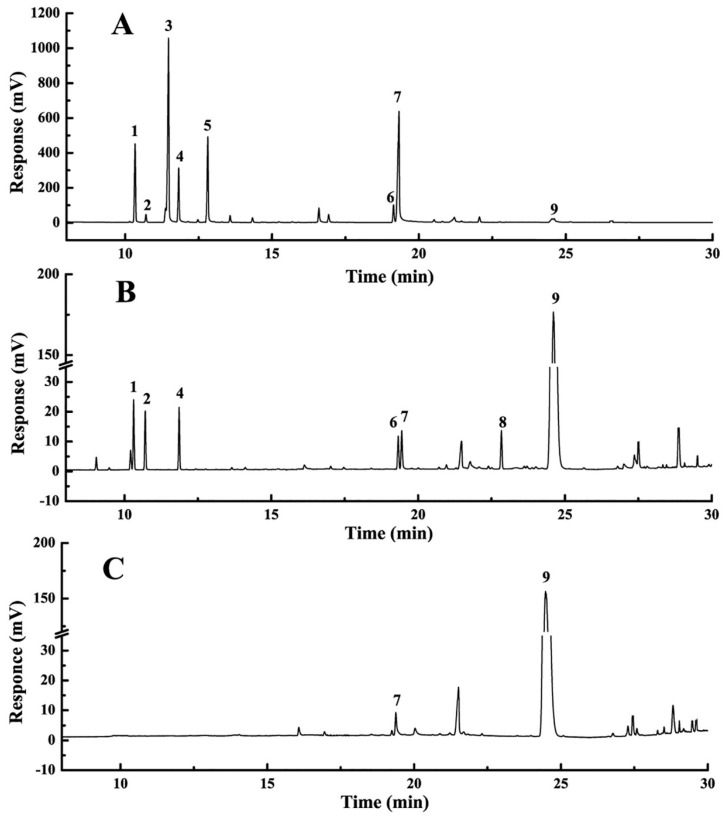
GC chromatograms of HEHEO (**A**); crude HEO (**B**); and pure HEO (**C**). Arabic numerals (1–9) marked in Figure 4 correspond to the compounds listed in Table 3.

Qualitative analysis was measured by both GC-MS and GC. Overall, there were nine compounds identified by comparing the obtained MS with those of the NIST library with matching degree ≥ 95% [36]. Then, hydrodistillation extracted HEO (HEHEO), crude HEO and pure HEO were further analyzed by GC. The GC chromatograms and corresponding results were shown in Figure 4 and Table 3, respectively. For the hydrodistillation extracted HEHEO (Figure 4A), it was mainly composed of α-pinene (10.24%), bicycle [3.1.0] hexane (30.87%), β-myrcene (12.60%) and 2-undecanone (22.63%). 2-undecanone was the oxidative product of Hou, however, the percentage of Hou was only 1.95% in HEHEO. For the extracts prepared by solvent extraction, the Hou percentage was 80.88% and 84.94% for crude HEO (Figure 4B) and pure HEO (Figure 4C), respectively. Contrastively, the percentage of 2-undecanone was less than 2%. It could be speculated that most of Hou was oxidized into 2-undecanone by the high temperature during the hydrodistillation extraction. Therefore, the solvent extraction used in this study had much higher efficiency than hydrodistillation extraction due to its mild extraction conditions and suitable solvent. 

**Figure 5:**
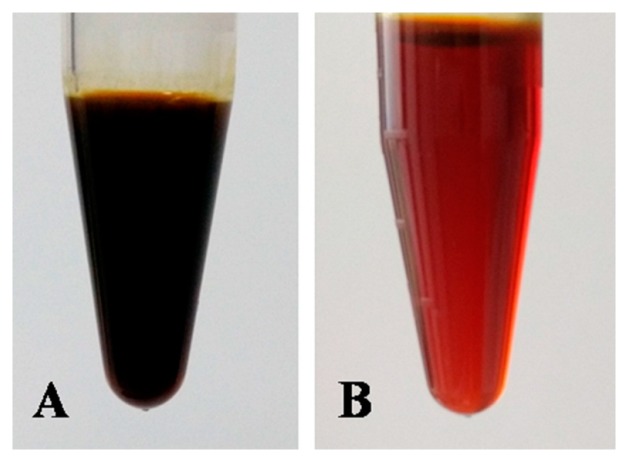
Photos of crude HEO (**A**) and pure HEO (**B**).

However, it was important to note that the Hou percentage in Table 3 was calculated by area normalization method, which was not suitable for Hou content determination. To correctly determine the Hou content, the standard curve method was used. Through calculation, the content of Hou was increased from 15.0% to 43.4% with the recovery of 58.4% after purification by D101 resin. Meanwhile, validation experiments revealed that the average Hou content in pure HEO was 44.3% ± 2.01%, which showed a great potential for industrial applications. Additionally, it was easy to find out that the pure HEO (Figure 5B) had a deep red color and transparent appearance after purification by D101 MR. Furthermore, it had a characteristic fishy odor and better liquidity, which represented a nice quality. Based on the above findings, it could be concluded that the method of solvent extraction following D101 purification to prepare Hou-riched HEO was highly efficient and could be further applied to large-scale preparation.

### 2.4. Characterization of HME

**Figure 6:**
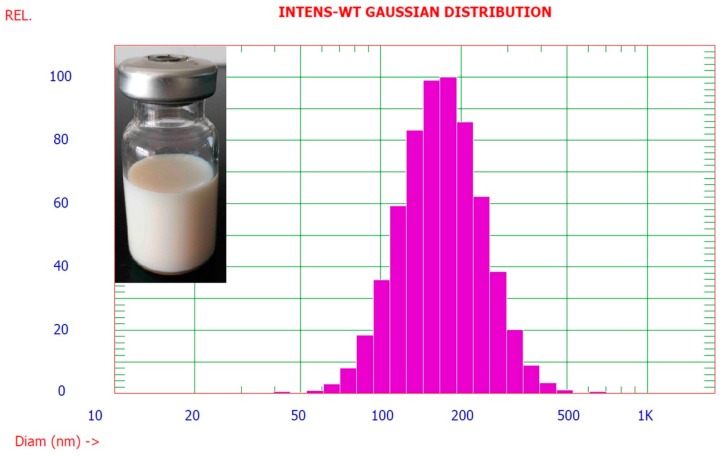
Photo of HME and its particle size distribution determined by the PSS NICOMP Particle Size System.

To further improve the safety and activities of HEO, HME (pure HEO: 2 mg/mL) was prepared and characterized. As shown in Figure 6, the prepared HME was a white milky liquid with a uniform appearance and no floating oil. The average particle size (Figure 6) and polydispersity index (PI) of HME were 179.1 nm and 0.126, respectively. HME had a high negative zeta potential of −37.51 mV with a high encapsulation rate of 94.7%. These results indicated that pure HEO was successfully encapsulated into ME and HME had excellent potential for industrial-scale production.

### 2.5. Safety Evaluation

#### 2.5.1. In Vitro Hemolysis Tests

It is reported that *H. cordata* injection could cause hemolysis due to the impurities of HEO or Tween 80 in the formulation [15,16]. In vitro hemolysis tests were carried out to compare the hemolytic effect of different extracts. As shown in Table 4, HEHEO had serious hemolytic effect and Tween 80 solution (2.5%) showed weak hemolysis, suggesting that HEHEO was toxic to erythrocyte. Compared with the crude HEO, pure HEO exhibited weak hemolysis that may result from Tween 80 in the solution, suggesting a reduced toxicity to erythrocyte after purification by D101. However, the HME, which was prepared by encapsulation of pure HEO in ME, showed no hemolysis or agglutination. This indicated that MEs could further improve the safety of pure HEO. Considering the high encapsulation rate of HME (94.7%), it could be speculated that pure HEO was fully encapsulated in the inner oil phase by the biodegradable and biocompatible shell. which could avoid the contact of HEO with erythrocyte and thus avoided hemolysis. Furthermore, the tests indicated that hemolysis induced by HEHEO was one of the important reasons for ADR. Over all, HEHEO could induce hemolysis, MR purification could reduce hemolysis, and MEs could avoid hemolysis effectively.

#### 2.5.2. Acute Toxicity Test

Acute toxicity test was performed to compare the safety of HEHEO, crude HEO, pure HEO, and HME. Briefly, mice were intravenously injected with Hou at the dose of 30 mg/kg and observed for toxic symptoms and mortality. As shown in Table 5, blank Tween 80 solution and blank ME had no effect on mice. HEHEO exhibited significant acute toxicity such as tachypnea, jitter, skip, ataxia, and tic, though no death occurred. For the crude HEO group, nine of the 10 treated mice died after administration from severe toxicity, such as tachypnea, dyspnea, tic, and fainting. For the pure HEO group, the mice showed tachypnea, ataxia and recovered within 3 min without death. However, there were no obvious reactions observed in the HME group. Therefore, we concluded that purification by D101 resin decreased the toxicity of HEO and ME encapsulation further improved its safety. In addition, the results indicated that the impurities in the crude HEO were seriously toxic and the purification by D101 resin could effectively remove the toxic impurities in the crude extract.

Both in vitro hemolysis test and acute toxicity test proved that the HEHEO extracted by hydrodistillation showed serious toxicity, whereas purification followed by ME encapsulation could effectively improve the safety of HEO.

### 2.6. In Vitro Antiviral Activity

The in vitro antiviral activities of HEHEO, crude HEO, pure HEO, and HME against CVB3 and B6 were evaluated by using the conventional CPE method [37]. As shown in Table 6, HEHEO exhibited weak activity against CVB3 with IC_50_ of 17.24 µg/mL. For the extracts prepared by solvent extraction, pure HEO (Hou 46.6%) showed significant antiviral activity with IC_50_ of 7.41 µg/mL while the crude HEO (Hou 15.0%) showed relatively weak activities (IC_50_ > 15 µg/mL). This indicated that the solvent extraction following purification by D101 resin largely increased the content of Hou and, thus, improved the antiviral activity of HEO. Furthermore, the IC_50_ of HME was 0.35 µg/mL and SI was no less than 519.5 on CBV6. It is worth mentioning that the SI of HME was at least 100 times higher than that of the pure HEO without ME encapsulation. The largely increased SI indicated that ME encapsulation could simultaneously improve the safety and antiviral activity. The reasons could be explained as follows: HEO was wrapped in the inner oil phase and was controlled and sustained release to the virus by the shell of HME, which prolonged the action time and, thus, improved the activity. Additionally, the biodegradable and biocompatible shell (composed of phospholipids and Poloxamer 188) avoided the contact of HEO with Vero cells effectively, thereby greatly reducing the toxicity of HEO (TC_50_ > 181.82 µg/mL) [38]. Furthermore, it was reported that nanocarriers with inherent immunogenic properties would improve its activity [39].

CVBs is one of the most commonly identified agents for infection that is associated with acute and chronic myocarditis. However, there is no approved vaccine or antiviral drug for the prevention or treatment of CVB-induced diseases to date [40]. HME exhibited strong antiviral activities against CVB3 and B6 with better safety, which may provide an important strategy for anti-CVB and myocarditis therapy.

## 3. Material and Methods

### 3.1. Materials and Animals

#### 3.1.1. Macroporous Resin and Solvents

D101 macroporous resin was supplied by Sinopharm Chemical Reagent Co. Ltd. (Beijing, China). HP-20, HPD100 and AB-8 resins were purchased from Solarbio (Beijing, China). All chemicals and solvents were of analytical grade and used without further purification.

#### 3.1.2. Plant Material

Fresh roots of *H. cordata* were obtained from Yizhou (Guangxi, China). The botanical identification of the plant material was performed by Renyun Wang from Institute of Materia Medica and a voucher sample was sealed and stored at −40 °C. Fresh roots were washed, dried in the dark and cut into pieces with appropriate length before extraction.

#### 3.1.3. Animals

Sixty ICR mice (20 ± 2 g) of both sexes and one male rabbit (2 kg) were purchased from Charles River (Beijing, China). Animals were allowed to acclimate to the laboratory environment for five days. The animal experiments were approved by the Animal Care and Welfare Committee of the Institute of Materia Medica, Chinese Academy of Medical Sciences and Peking Union Medical College.

### 3.2. Analysis of Extract

#### 3.2.1. Qualitative Analysis by GC-MS 

The GC-MS system was composed of an Agilent 7890A series GC connected to an Agilent 5975C mass selective detector. An Agilent HP-5MS capillary column (30 m × 0.25 mm, 0.25 μm, Agilent Technologies, Santa Clara, CA, USA) was used for separation. Ultrapure helium was used as the carrier gas at a flow rate of 1.0 mL/min with injector temperature of 250 °C. One microliter of sample was injected under split mode (ratio 1:20). The oven temperature program was 50 °C for 2 min, rising at 4 °C/min to 150 °C for 3 min, followed by rising at 20 °C/min to 300 °C then held for 10 min. Electronic ionization source and quadrupole temperatures were 230 °C and 150 °C, respectively. All mass spectra were recorded in the full scan mode at 70 eV (*m*/*z* 20–800 amu) and the identifications of the compounds were conducted by comparing the obtained mass spectra (MS) with those of the NIST library.

#### 3.2.2. Quantitative Analysis of HEO by GC

An Agilent 6890N series GC system equipped with a hydrogen flame ionization detector (FID) was used for quantitative analysis. Separation was achieved with an Agilent DB-5 capillary column (30 m × 0.25 mm, 0.25 μm, Agilent Technologies, Santa Clara, CA, USA). The carrier gas was nitrogen with a flow of 1.0 mL/min. The oven temperature was programmed at 50 °C for 3 min, with a rise of 5 °C/min up to 150 °C and held for 3 min, followed by rising at 20 °C/min to 250 °C, and held for 5 min. The temperature of both the injector and detector was set at 250 °C. One microliter of sample was injected under split mode (ratio 1:3). External standard calibration was used for the Hou quantitative analysis. A series of Hou standards in the range of 4.32–216 μg/mL were prepared in ethyl acetate solution to obtain the linear response (*Y* = 6.587*X* − 5.367, r = 0.9999), where Y was the peak area of Hou, and X was the concentration of Hou (μg/mL).

### 3.3. Preparation of HEO

#### 3.3.1. Preparation of Hydrodistillation Extracted HEO (HEHEO)

HEHEO was obtained by hydrodistillation of 300 g of fresh roots (cut into about 1 cm pieces) of *H. cordata*, with 0.5 L of distilled water for 3 h, using a Clevenger apparatus [10]. Then, ethyl acetate was used to wash the oil and then removed by a rotary evaporator. The HEHEO was stored in sealed vials protected from light at 4 °C for further analysis.

#### 3.3.2. Optimization for Solvent Extraction of Crude HEO 

A L9 (3) ^4^ orthogonal experimental design was used to optimize four variables (ultrasonic time, extraction time, root length, and solid-liquid ratio) with the extraction ratio as the index. The factors and levels of individual variables were presented in Table 1. The experimental design was presented in Table 2, along with experimental data. The influence of the factor was determined by calculating the range of the k, which was the mean ratio of the corresponding level. The optimized extraction process was used for further extraction. The extract was further concentrated under reduced pressure at 40 °C using a rotary evaporator to remove the solvents. Next, the concentrated extract was dispersed in ethanol and the precipitate was removed by centrifugation. Finally, the product was extracted by *n*-hexane. After removing *n*-hexane, the crude HEO was obtained and stored at −20 °C.

### 3.4. Optimization for Purification of HEO by MR

#### 3.4.1. Static Adsorption-Desorption Tests to Optimize MR Types

Four types of MRs (D101, HP20, AB-8 and HPD100) were used in this study. The resins were pretreated by 95% ethanol for 24 h followed by deionized water to remove the monomers and porogenic agents trapped inside the pores during the synthesis process. Prior to use, the resins were wetted with ethanol and then washed with deionized water until the ethanol was thoroughly replaced. The moisture contents of the tested resins were determined by drying the beads at 100 °C to a constant weight in an oven. The moisture contents of D101, AB-8, HPD100 and HP20 resins were 52.12%, 62.11%, 72.17%, and 57.67%, respectively.

In order to select the most suitable MR to purify the crude HEO, the adsorption and desorption properties of the four resins were characterized. One gram of each resin (dry) was placed into a conical flask and 20 mL sample solution (crude HEO 5.0 mg/mL) was added. The flask was then shaken in a shaker (120 rpm) for 12 h at 25 °C. After reaching the adsorption equilibrium, the sample solution was removed and the Hou content was analyzed by GC. Then, the resin was washed with deionized water and desorbed with 10 mL 80% ethanol (*v*/*v*), then shaken (120 rpm) for 12 h at 25 °C. The Hou content in desorption solution was analyzed. The adsorption capacity, desorption capacity and desorption ratio of each resin were selected as indexes and calculated by the following formulas:

(1) $$\text{Adsorption capacity}=\frac{V_{0}\times \left(C_{0}-C_{e}\right)}{W}$$

(2) $$\text{Desorption capacity}=\frac{V_{d}C_{d}}{W}$$

(3) $$\text{Desorption ratio}=\frac{V_{d}C_{d}}{V_{0}\times \left(C_{0}-C_{e}\right)}\times 100\%$$

where adsorption capacity (mg/g) represented the amount of Hou adsorbed on 1 g of dry resin; desorption capacity (mg/g) represented the amount of Hou after adsorption equilibrium; C_0_ and C_e_ were the initial and equilibrium concentrations of Hou (mg/mL), respectively; W was the weight of the dry resin (g); V_0_ and V_d_ were the volumes of adsorption and desorption solutions (mL), respectively; and C_d_ represented the concentration of Hou in the desorption solution (mg/mL).

#### 3.4.2. Dynamic Adsorption-Desorption Tests to Optimize Purification Conditions

Dynamic adsorption-desorption experiments were performed in a glass columns (1.5 cm × 20 cm) (Tianjin Tianbo Glass Instrument Co. Ltd., Tianjin, China) wet packed with 12 g (wet resin) of the D101 resin. The bed volume (BV) of the resin was 20 mL. After loading the sample, the column was washed with distilled water (2 BV) and ethanol–water (30:70, 5 BV) to remove impurities and then desorption solvents. The conditions were as follows: Hou concentration of the loading sample solution was 0.225 mg/mL; sample flow rate was 6 BV/h, desorption solvents included different ethanol/water solutions (60:40, 70:30, 80:20, 90:10, and 95:5) with an adsorption flow rate of 6 BV/h.

### 3.5. Preparation and Characterization of HME

HME was prepared by high-pressure homogenization as previously reported by our team [29]. The HME particle size and zeta potential were measured by a PSS NICOMP Particle Size System (PSS, Port Richey, FL, USA) after dilution with double-distilled water. The encapsulation rate of HME was determined by measuring the free Hou in the aqueous phase through the high-speed centrifugation (Beckman XL-90, Beckman Fullerton, CA, USA) of 40,000× *g* for 8 h at 4 °C.

### 3.6. Safety Evaluation

#### 3.6.1. In Vitro Hemolysis Test

About 10 mL of blood was collected from a rabbit and centrifuged at 395× *g* for 10 min to remove the supernatant. The precipitated blood cells were then washed with 0.9% sodium chloride several times until the supernatant was colorless. At last, an erythrocyte suspension (2%, *v*/*v*) was prepared by suspending the erythrocyte into the 0.9% sodium chloride.

HEHEO, crude HEO, and pure HEO were dispersed in Tween 80 solution (2.5%) to prepare samples with the extract concentration of 2.0 mg/mL, respectively. Tubes 1–5 were filled with 0.5, 0.4, 0.3, 0.2, and 0.1 mL of sample, respectively. Then, 2.5 mL of 2% erythrocyte suspension (*v*/*v*) and different amounts of 0.9% sodium chloride were added to make a total volume of 5 mL. For the tubes 6% and 7%, 0.9% sodium chloride and distilled water replaced the sample solutions as blank control and positive control, respectively. Then, the tubes were placed into a 37 °C water bath with gentle shaking. The results were recorded at 30 min as well as at 1 h, 2 h, 3 h, and 4 h. The results were interpreted qualitatively as follows:

Hemolysis (++): transparent and red solution without erythrocyte sinking.
Weak hemolysis (+−): light red or brown solution with a small amount of erythrocyte sinking.
No hemolysis (−−): erythrocyte sink leaving a colorless and clear supernatant.
Agglutination (Agg.): dark brown or reddish flocculent precipitate in the solution.

#### 3.6.2. Acute Toxicity Test

Sixty mice were randomly assigned to six groups of ten animals each (five males and five females). Before the test, crude HEO and pure HEO were dispersed in Tween 80 solution (2.5%) to prepare samples with the Hou concentration of 1.5 mg/mL, respectively. The crude HEO, pure HEO and HEM (Hou 1.5 mg/mL) were injected through the caudal vein with Hou at the doses of 30 mg/kg. For the HEHEO group, the extract was dissolved in Tween 80 solution (2.5%) with the extract concentration of 4 mg/mL. Negative controls were blank Tween 80 (2.5%) solution and blank ME. Additionally, no-treatment control was set for observation of symptoms and insurance of accuracy. General behaviors of mice and symptoms of toxicity were observed continuously for 24 h after injection.

### 3.7. In Vitro Antiviral Activity

#### 3.7.1. Cells and Viruses

African green monkey kidney cells (Vero) were purchased from the American Type Culture Collection (ATCC, Manassas, VA, USA) and cultured in MEM supplemented with 10% FBS and antibiotics (100 U/mL penicillin G, 100 μg/mL streptomycin) at 37 °C in a humid atmosphere (5% CO_2_ −95% air). Coxsackie Virus B3 (CVB3, strain Nancy) and B6 (CBV6, strain Schmitt) were obtained from ATCC and propagated in Vero cells.

#### 3.7.2. Cytotoxicity Assay

The cytotoxicity effects of the samples (HEHEO, crude HEO, pure, and HME) toward Vero cells were monitored by the MTT assay. Vero cells seeded in 96-well plates were incubated with various concentrations of tested samples or ribavirin and pleconaril (positive controls) for 72 h at 37 °C. The cells were then incubated with 20 µL MTT reagent (5 mg/mL, Sigma-Aldrich, St. Louis, MO, USA) for 4 h at 37 °C. The MTT reagent was removed and 150 µL DMSO was added to each well. The spectrophotometric reading was taken at 490 nm using a microplate reader (Perkin-Elmer, Waltham, MA, USA). 5Fifty percent (50%) toxic concentration (TC_50_) was defined as the concentration that inhibited 50% cellular growth in comparison with the untreated controls and calculated using the Reed-Muench method.

#### 3.7.3. Antiviral Activity

The antiviral activity against CVB3 and CVB6 was evaluated using the cytopathic effect (CPE) reduction method. Briefly, cells were plated into 96-well culture plates and incubated for 24 h. The medium was then removed and cells were infected with virus for 2 h. Samples with different various concentrations (HEHEO, crude HEO, pure HEO, and HME) were added to each well immediately and incubated until the CPE of the control group cells reached 4. The 50% inhibitory concentration (IC_50_) was determined using the Reed-Muench method. The selective index (SI) was calculated from the ratio of TC_50_/IC_50_.

## 4. Conclusions

In this study, pure HEO with high Hou content (43.4%) was prepared by solvent extraction followed by purification using D101 resin. To further improve its safety and antiviral activity, the pure HEO was encapsulated into ME and then comprehensively characterized. The safety evaluation tests and in vitro antiviral test indicated that the safety and antiviral activity of HEO were largely improved after purification by D101 resin and were further improved by ME encapsulation. These results indicated that purification of HEO using D101 resin, followed by ME encapsulation, was of high efficiency, reduced toxicity, and enhanced effect, which was very promising for future therapy of antiviral and infectious diseases. Hou, the major active constituent of HEO, possesses a quite simple chemical structure and high antiviral activities. This is straightforward to evaluate its usefulness as a pure compound in the future.

## Figures and Tables

**Table 1:** Factors and levels of orthogonal experimental design.

Levels	Factor			
	A (Ultrasonic Time, min)	B (Extraction Time, day)	C (Root Length, cm)	D (Solid-Liquid Ratio, kg/L)
1	0	1	no cutting	1/1
2	20	2	5	1.5/1
3	40	3	1	2/1

**Table 2:** Results of the L9 (3) ^4^ orthogonal tests.

Number	A (Ultrasonic Time, min)	B (Extraction Time, Day)	C (Root Length, cm)	D (Solid-Liquid Ratio, kg/L)	Extraction Ratio (%)
1	1	1	1	1	0.070
2	1	2	2	2	0.114
3	1	3	3	3	0.152
4	2	1	2	3	0.122
5	2	2	3	1	0.120
6	2	3	1	2	0.178
7	3	1	3	2	0.112
8	3	2	1	3	0.128
9	3	3	2	1	0.068
*k_1_*	0.112	0.101	0.125	0.086	
*k_2_*	0.140	0.121	0.101	0.135	
*k_3_*	0.103	0.133	0.128	0.134	
*R*	0.037	0.032	0.027	0.051	

Note: *k_i_* = $\frac{\sum^{\text{​}}i}{3}$: the mean extraction ratio of the corresponding level of each factor; *R* = max{*k_i_*} − min{*k_i_*}: the range of the *k_i_* of each factor.

**Table 3:** GC analysis of HEHEO, crude HEO and pure HEO with peak identification and area percentage.

No.	Compounds	HEHEO		Crude HEO		Pure HEO	
		RT (min)	(%) ^a^	RT (min)	(%) ^a^	RT (min)	(%) ^a^
1	α-pinene	10.315	10.24	10.303	2.58	\	\
2	Camphene	10.686	1.05	10.690	2.17	\	\
3	Bicycle [3.1.0] hexane	11.473	30.87	\	\	\	\
4	β-pinene	11.806	7.75	11.883	2.35	\	\
5	β-myrcene	12.853	12.60	\	\	\	\
6	Bornyl acetate	19.157	2.57	19.390	1.37	\	\
7	2-undecanone	19.303	23.63	19.512	1.71	19.382	1.36
8	Caryophyllene	\	\	22.846	1.67	\	\
9	Houttuynin	24.559	1.95	24.691	80.88	24.468	84.94

Note: RT: Retention time; ^a^: Relative percentage obtained from peak area; HEHEO: hydrodistillation extracted *H. cordata* essential oil; crude HEO: *H. cordata* essential oil extracted by solvent extraction; pure HEO: crude HEO purified by macroporous resin.

**Table 4:** In vitro hemolysis test results of HEHEO, crude HEO, pure HEO, and HME.

Samples	Tube Number						
	1	2	3	4	5	6	7
Tween 80 solution (2.5%)	+−	+−	+−	−−	−−	−−	++
Blank ME	−−	−−	−−	−−	−−	−−	++
HEHEO	++ *	++ *	++ *	++	++	−−	++
Crude HEO	++	++	+−	+−	+−	−−	++
Pure HEO	+−	+−	+−	+−	+−	−−	++
HME	−−	−−	−−	−−	−−	−−	++

Note: ++: Hemolysis; +−: Weak hemolysis; −−: No hemolysis; *: Erythrocytes are hemolytic in 1 h.

**Table 5:** Acute toxicities of HEHEO, crude HEO, pure HEO, and HME in mice.

Sample	Symptoms	Mortality (D/T)	Mortality (%)
Tween 80 solution (2.5%)	No obvious reactions	0/10	0
Blank ME	No obvious reactions	0/10	0
HEHEO	Tachypnea, jitter, skip, ataxia, tic and recovered within 5 min	0/10	0
Crude HEO	Tachypnea, dyspnea, tic and fainting	9/10	90
Pure HEO	Tachypnea, ataxia and recovered within 3 min	0/10	0
HME	No obvious reactions	0/10	0

Note: D/T, Dead/Treated mice.

**Table 6:** In vitro antiviral activities of HEHEO, crude HEO, pure HEO, and HME against CVB3 and B6.

Samples	CVB3			CVB6		
	TC_50_ (µg/mL)	IC_50_ (µg/mL)	SI	TC_50_ (µg/mL)	IC_50_ (µg/mL)	SI
HEHEO	72.09	17.24	4.18	72.09	>22.22	\
Crude HEO	75.28	22.22	3.1	75.28	15.41	4.5
Pure HEO	53.44	7.41	5.4	53.44	7.41	5.4
HME	>181.82	>0.82	\	>181.82	0.35	>519.5
Pleconaril	15.41	0.0007	22,014.3	15.41	0.037	416.5
Ribavirin	2000	292.46	6.8	2000	384.88	5.2
